# Barriers to and motivators of handwashing behavior among mothers of neonates in rural Bangladesh

**DOI:** 10.1186/s12889-018-5365-1

**Published:** 2018-04-11

**Authors:** Shahana Parveen, Sharifa Nasreen, Jelena V. Allen, Kelly B. Kamm, Shifat Khan, Shirina Akter, Tajnin Marin Lopa, K. Zaman, Shams El Arifeen, Stephen P. Luby, Pavani K. Ram

**Affiliations:** 1 Programme for Emerging Infections, Infectious Diseases Division, icddr,b (International Centre for Diarrhoeal Diseases Research, Bangladesh), 68, Shaheed Tajuddin Ahmed Sarani, Mohakhali, Dhaka, 1212 Bangladesh; 2 Maternal and Neonatal Health, Maternal and Child Health Division, icddr,b, 68, Shaheed Tajuddin Ahmed Sarani, Mohakhali, Dhaka, 1212 Bangladesh; 3 School of Public Health and Health Professions, University at Buffalo, 3435 Main Street, Rm. 237 Farber Hall, Buffalo, NY 14214 USA; 4 Infectious Diseases and Geographic Medicine, Stanford University, Y2E2, MC 4205473 Via Ortega, Stanford, CA 94305 USA

**Keywords:** Handwashing, Maternal, Neonatal, Bangladesh

## Abstract

**Background:**

To design a maternal handwashing intervention for the newborn period, this qualitative study explored drivers of handwashing among mothers and other caregivers of neonates and infants in two rural areas of Bangladesh.

**Methods:**

We conducted 40 semi-structured observation sessions to observe handwashing behaviors of primiparous and multiparous mothers of neonates, and to understand the contextual factors that facilitated or hampered those behaviors. We then conducted 64 interviews with mothers of neonates and mothers of infants and 6 group discussions with mothers of infants, other female caregivers and fathers to explore perceptions, beliefs, and practices related to handwashing in the neonatal period. Based on a conceptual model and the Theory of Reasoned Action/Theory of Planned Behavior, we developed a conceptual model a priori*,* we performed thematic analysis to explain determinants of maternal handwashing behaviors.

**Results:**

We conducted 200 h of observation among mothers of neonates. The age range of participating mothers varied between 17 and 25 years and their maximum education was up to 10th grade of schooling. Mothers, other female caregivers and fathers perceived a need to wash hands with or without soap before eating or before feeding a child by hand to prevent diarrhea. Mothers expressed the importance of washing their hands before holding a baby but were rarely observed doing so. All respondents prioritized using soap for visible dirt or feces; otherwise, water alone was considered sufficient. Lack of family support, social norms of infrequent handwashing, perceptions of frequent contact with water as a health threat and mothers’ restricted movement during first 40 days of neonate’s life, and childcare and household responsibilities adversely impacted handwashing behavior.

**Conclusions:**

Addressing emotive drivers of handwashing within existing social norms by engaging family members, ensuring handwashing facilities and clarifying neonatal health threats may improve maternal handwashing behavior in the neonatal period.

**Electronic supplementary material:**

The online version of this article (10.1186/s12889-018-5365-1) contains supplementary material, which is available to authorized users.

## Background

In high neonatal mortality settings, an estimated 44% of neonatal deaths occur because of infectious syndromes such as sepsis, acute respiratory infection, neonatal tetanus, and diarrhea [1]. Hand hygiene could reduce transmission of pathogens from caregivers’ hands to neonates during birth as well as childcare [2]. Promoting handwashing to mothers in the post-neonatal period reduces the risk of acute respiratory infection and diarrhea among infants > 28 days old [3, 4]. In a secondary data analysis from a cluster-randomized trial in Nepal, Rhee and colleagues found that neonates delivered by birth assistants who reported washing their hands and whose mothers reported washing their hands during the first 14 days experienced fewer umbilical cord infectious compared to the participants who reported not washing their hands [5]. Furthermore, neonatal mortality was significantly lower among children whose mothers reported washing their hands before handling their children compared to children whose mothers did not report washing their hands [6]. Another randomized controlled trial evaluating the effect of handwashing with soap as a lone intervention and along the with application of 4% chlorhexidine on umbilical cords did not report any significant effects of handwashing on umbilical cord infections [RR = 0·83, 95% CI: (0·61–1·13), *p* = 0·24] or neonatal mortality (RR = 1.08, 95% CI: (0.79, 1.48), *p* = 0.62] compared to control [7]. The possible explanations for the lack of effect impact could either be that actual handwashing practices differed (typically lower) from reported behaviors or that the application of different traditional materials on neonatal umbilical cords continued.

According to the 2014 Bangladesh Demographic and Health Survey (BDHS), the neonatal mortality rate was 28/1000 live births that comprised 61% of all under-five deaths [8]; and in 2012, 40% of deaths were attributed to pneumonia, sepsis and diarrhea [9]. Previous studies in Bangladesh have largely focused on the handwashing behaviors of mothers of children under the age of 5. Some of these studies have reported infrequent handwashing among mothers and other caregivers of young children at times of possible pathogen transmission, including after contact with fecal matter, typically the most frequently reported time that required hands to be washed [10, 11]. The perceptions, beliefs and practices related to maternal handwashing behavior in the neonatal period differs from those of mothers with older children, as observed by Greenland and colleagues in Indonesia [12] but there is limited evidence in this regard from Bangladesh. Such detailed understanding of behavioral drivers is important for developing a maternal handwashing intervention to reduce the risk of neonatal morbidity and mortality attributed to hygiene-preventable causes. Nested in a larger experimental study whether perinatal handwashing promotion results in reduced neonatal morbidity, we sought to explore current handwashing practices and the context of barriers and motivators to maternal and caregiver hand hygiene that could aid in designing target maternal handwashing intervention in Bangladesh specific to the neonatal period.

## Methods

### Study site, timeline, and sampling

This qualitative study was conducted in the rural areas of Matlab sub-district and Habigonj district in Bangladesh among mothers of neonates and mothers of infants. In rural Bangladesh, women’s social status is typically predominantly linked to traditional social and cultural norms that sometimes limit women’s autonomy in decision making or accessing specific health services [13]. Therefore, we included secondary female caregivers in the family and fathers of neonates or young infants in the study as they are important family decision-makers.We followed both random and chain referral sampling for respondent selection in the study sites.

#### Site-specific description and methods: Matlab

*Matlab* is a rural sub-district of Chandpur district in south-eastern Bangladesh where icddr,b (formerly International Centre for Diarrhoeal Diseases Research, Bangladesh) maintains a Health and Demographic Surveillance System (HDSS), with added maternal and child health (MCH) interventions in some villages. We conducted this study in 18 villages where the MCH interventions did not occur and the rate of institutional delivery was similar to other rural areas in Bangladesh [Table 1] [9, 14]. From October 2010 to February 2011, we collected data among four groups of primiparous- i) mothers of neonates, ii) mothers of infants, iii) other female caregivers in the family (typically elders and maternal or paternal grandmothers of neonates) and iv) fathers. We created a list of mothers of neonates less than 28 days old and mothers of infants less than 1 year old from the Matlab HDSS pregnancy database. From this list, we randomly selected mothers who met the criteria mentioned above. In this site, we selected primiparous mothers of both neonates and infants with the understanding that during a woman’s first pregnancy, her risk perception and other emotion-based behavioral responses may be enhanced and might encourage a new mother to adopt a new behavior [15]. In addition, we also selected mothers, whose children were at the infant stage since they had already experienced the neonatal period, and hence, might be more likely to offer information regarding both the neonatal period and later infancy. Secondary female caregivers (e.g. elders and maternal or paternal grandmothers of neonates) in the family, played influential role for neonatal care and fathers of primiparous neonates and infants were identified based on recommendation by mothers (i.e. chain referral sampling).We excluded the mothers who lived in the study areas but temporarily migrated to their parents’ homes outside the study areas for delivery of the newborn.

**Table 1:** Key indicators of neonatal and maternal health in Matlab and Habigonj (under Sylhet division), Bangladesh [9, 14]

Indicator		Matlab (2011 HDSS)	Sylhet division^a^ (2011 BDHS)
Neonatal mortality rate (per 1000 live births)		22 (government service area)	45
Infant mortality rate (per 1000 live births)		28 (government service area)	59
Institutional delivery		23% (government service area)	21%
Birth attended to by traditional birth attendants (trained and untrained)		46% (government service area)	72%
Female literacy rate		30%	17%
Religion	Muslim	90%	80%
	Hindu	9%	19%
Geographic distribution		Low-land agro-based area	High-land area

^a^Bangladesh Demographic and Health Survey (BDHS) report refers national level representative data that includes only division level data, not the district level data

#### Site-specific description and methods: Habigonj

We also collected data from 16 villages in that *Habigonj* district under the Sylhet division in the north-eastern part of Bangladesh (Table 1) from September to December, 2011. The population of Habigonj is typically conservative; women have limited access to healthcare services, and conservative Muslim religious groups strongly influence various norms of childcare [16]. Similar to the method used in Matlab, we generated a list of mothers of neonates and infants from the existing demographic surveillance database from an ongoing project on Integrated Safe Motherhood, Newborn Care, and Family Planning, called MaMoni, which was funded by the United States Agency for International Development (USAID). This project promoted various community-based approaches to safe motherhood and reducing neonatal mortality [17]. In this site, we selected both primiparous and multiparous mothers of neonates and mothers of infants, allowing us to compare the differences in behaviors related to childcare and hand hygiene among mothers with single and multiple children. We excluded mothers who received any intervention from MaMoni related to handwashing. We also sought information from mothers of infants, other female caregivers, and fathers using the chain referral sampling methods similar to those applied in Matlab.

### Data collection and study participants

In each study area, a team of four qualitative researchers with training in anthropology collected data. In each site we collected data using specific guidelines tailored for several qualitative data collection tools [see Table 2]. The research team received training on qualitative data collection methods and study-specific guidelines with pre-testing prior to the data collection.

**Table 2:** Data collection tools and sample from Matlab and Habigonj, Bangladesh, 2010–2011

Data collection tools	Type of participant	Matlab (Primiparous mothers)	Habigonj (both primiparous and multiparous mothers)	Issues observed/explored
Observation (*n* = 40)	Mother of neonate < 28 days	20	20	Recorded several handwashing opportunities; handwashing opportunities were defined as critical times (before feeding or touching baby, after cleaning child’s bottom and after visiting the toilet) of possible pathogen transmission to or from mother’s hands. We recorded mother’s practices at these handwashing opportunities of the pre defined critical events and try to understand the contextual factors that facilitated or hampered their handwashing behaviors. During the sessions, we also recorded if anyone other than mothers touched or hold the child and their relevant handwashing behavior before the event
In-depth interview (*n* = 64)	Mother of neonate < 28 days	20	20	Explored perceptions, beliefs, and local practices related to handwashing
	Mother of infant > 28 days to < 12 months old	12	12	
Group discussion (*n* = 6)	Mother of infant > 28 days to < 12 months old	1 (9 participants)	1 (6 participants)	Discuss issues related to childcare and hand hygiene in their area
	Female caregivers other than mothers	1 (10 participants)	1 (6 participants)	
	Father of neonates or young infants ≤6 months old	1 (6 participants)	1 (6 participants)	

i) Semi-structured observations

We conducted single 5-h semi-structured observation sessions (from 8 am to 1 pm) in households of mothers of neonates to observe their typical activities. Mothers were informed that the major objective was to observe their daily practices and routines of feeding, cleaning and overall caring behaviors for their neonates. In a semi-structured observation form, we recorded events of potential pathogen transmission and whether or not mothers washed their hands with or without soap at those opportunities. Events of potential pathogen transmission included feeding or touching the baby, cleaning a child’s bottom, mothers eating and going to the toilet. We also took detailed handwritten notes of the physical environment and setting of each event to understand the possible contextual factors that could facilitate or hamper handwashing behavior, such as mothers involved in multiple tasks, no help from attendants etc. [6]. In subsequent in-depth interviews with the same mothers of neonates, respondents later confirmed some factors documented as facilitators or barriers for handwashing during observation sessions.

- ii) In-depth interviews

We conducted in-depth interviews with both mothers of neonates and mothers of infants to elucidate their perceptions and opinions about the health of their children, healthy practices, prevention of illness, and taking care of children. We interviewed the same mothers of neonates whom we also observed. After each observation session, we asked mother of neonate for appointment for an interview within the following two days. All the interviews were conducted in native Bengali in a private setting chosen by the informants and each lasted 60–90 min.

iii) Group discussions

After completing all the observation sessions and in-depth interviews, we organized group discussions to further explore the themes related to childcare and hand hygiene that emerged from the observations and in-depth interviews. In both sites, we conducted group discussions of 90–120 min duration with three different groups: mothers of infants, female caregivers other than the mother, and fathers of neonates or young infants. We did not conduct any group discussion with mothers of neonates since we assumed that they might wish to confine themselves to their household compounds, given the social norm of restricted movement outside the household during the postnatal period in rural areas of Bangladesh [18, 19] [For more details see Table 2].

### Data analysis

**Fig. 1:**
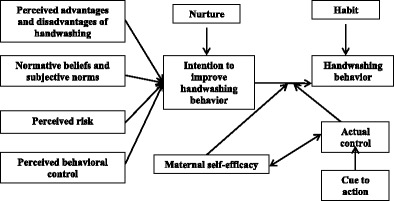
Conceptual model to explain motivations of maternal handwashing behavior in the neonatal period

Based on the Health Belief Model and the Theory of Reasoned Action/Theory of Planned Behavior, we developed a conceptual model a priori to explain determinants of maternal handwashing behavior in the neonatal period [Fig. 1]. As elucidated by McBride et al., pregnancy may represent a “teachable moment” because it is a time of enhanced risk perception, increased outcome expectancies, emotion-based behavioral responses, and a transforming vision of one’s role [15]. In her new role of motherhood, the woman undergoes a substantial change in her self-definition of her social role, as well as expectations of herself, and expectations regarding the impact of her behaviors on her child [20]. In contrast, multiparous women’s visions of their own roles may be far more fixed than those of primiparous women and, thus, they may be less “teachable” than primiparous women [15].We thus explored the behavioral determinants related to hand hygiene among both groups. Based on the conceptual model we primarily developed the guidelines for semi-structured observation (see observation guideline in Additional file 1) and in-depth interview.

To analyze the data from the semi-structured observations, the same research team expanded field notes taken during observations. We also counted the total handwashing opportunities at critical times and calculated the proportion of handwashing events occurred. We transcribed all audio recorded interviews and discussions verbatim in Bengali. Then we developed a code list based on the conceptual model and data collected, and coded all the Bengali transcriptions using the qualitative text organizing software Altas.ti 5.2.We performed thematic analysis of data from mothers, secondary caregivers, and fathers according to the themes identified in the conceptual model to describe perceptions related to hand hygiene behaviors, physical environment to perform required handwashing, and social dimensions that promote execution of mothers’ intended actions. We identified common trends and patterns from the responses and prepared summaries of each theme. We also compared the common or specific practices and driving factors to perform handwash that recoded in the observations with mothers of neonates and also reported by the same mothers. The comparisons allowed us to triangulate the data and confronted the evidences with the reporting.

## Results

### Description of demographic information of mothers in both sites

**Fig. 2:**
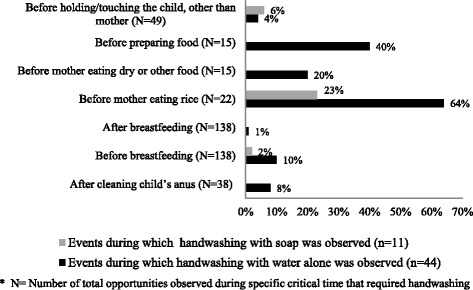
Performed handwashing within observed opportunities at critical times related to childcare (those observed > 10 times) among mothers of neonates/other caregivers in Matlab, Bangladesh, 2010–2011

**Fig. 3:**
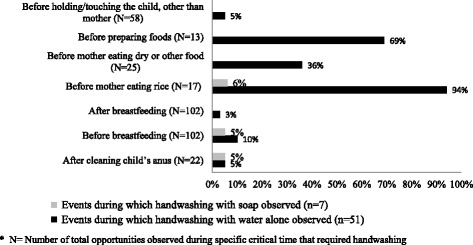
Performed handwashing within observed opportunities at critical times related to childcare (those observed > 10 times) among mothers of neonates/other caregivers in Habigonj, Bangladesh, 2011

Among 64 mothers we interviewed in both study sites, all were married; the age of primiparous mothers ranged from 17 to 22 years and the median age for multiparous mothers was 25 years (Table 3). In total, we observed mothers of neonates in both sites for 200 h, with 792 opportunities for handwashing [Figs. 2 and 3].

**Table 3:** Demographic information of the mothers enrolled for observations and in-depth interviews in Matlab and Habigonj, Bangladesh, 2010–12

		Matlab		Habigonj	
	Characteristics	Mothers of neonates (*n* = 20)	Mothers of Infants (*n* = 12)	Mothers of neonates (*n* = 20)	Mothers of Infants (*n* = 12)
Age of mother	Median age (IQR)	20 (19–22 years)	21.5 (20–24 years)	20 (18–24 years)	21 (19–24 years)
	Median age (IQR) (both neonates & infants)	20 (19–22 years), Primi (*n* = 32)		18 (17–20 years), Primi (*n* = 18) 25 (21–28 years), Multi (*n* = 14)	
Mother’s marital status	Married	100%		100%	
Sex of the child	Female	12	9	10	3
	Male	8	3	10	9
Age of the child	Median age (IQR)	15.5 days (14–24 days)	6.9 months (4–10 months)	12.5 days (9–17 days)	5 months (4–8 months)
Parity of mothers	Primi	20	12	10	8
	Multi	0	0	10	4
Type of delivery	Normal	16	10	19	11
	C-section	4	2	1	1
Delivery place	Delivery at parents’ home	8	6	5	2
	Delivery at marital home	2	1	11	5
	Delivery at facility	10	5	4	5
Mother’s residence during interview	Marital home	9	8	14	11
	Maternal home	11	4	6	1
Education of mothers	No education (0)	0	1	4	1
	≤ Class 5	6	3	15	4
	Class 6–10	11	6	1	7
Household income	Median income (IQR)	(*N* = 25)^a^ US$ 128 (US$ 64–128)		(*N* = 30)^b^ US$ 71(US$ 59–128)	

^a^Seven mothers in the Matlab site could not provide any specific information about their household income. Two of them had their own agricultural land, got crops (rice) yearly from those lands. These mothers were unable to provide approximate amount of equivalent cash income or expenditure

^b^Two mothers in the Habigonj site did not know about the monthly income or expenditure of their family

In both sites, typically the cooking area and toilet was separated from the bedrooms or living rooms of the main household, such as in one corner of yard. The handwashing station was generally situated in the outside of toilet. Usually, soap was not observed to keep in the common handwashing area shared by multiple families, but for single household, sometimes soap was observed available by the handwashing station.

### Maternal hand hygiene behaviors, as reported and observed

The hand hygiene behavior is a complex issue, combination of multiple overlapping factors. The maternal hand hygiene behaviors often influenced by her individual’s perceptions, physical environmental, or multiple social determinants to execute required hand hygiene:

#### Handwashing behavior

In in-depth interviews and group discussions, respondents described that their typical handwashing practice involves the use of water alone, generally without mention of soap. Respondents reported typically washing their hands, face and feet at least twice a day to clean themselves, after waking up in the morning and after finishing daily household chores or after returning home from daily work (for fathers) in the evening. Most mothers and other caregivers stated that they usually wash their hands if they are involved in cooking, sweeping, cutting vegetables or fish, or when hands are ‘visibly dirty’ (this refers to contact with mud, sand, dust, oily substances, soot, cooking spices, biomass fuel, and if there is contact with own feces, child and animal feces). Respondents perceived that after cutting when they washed vegetables, fish, or meat with water alone, or washing utensils or clothes, their hands were also being washed and thus did not need to be washed again before cooking. In addition, fathers reported that they washed their hands with soap after handling cow dung as such contact is considered profane; they also reported washing hands with soap after agricultural work in the field, such as harvesting or spraying pesticide. According to all types of respondents, hands need to be washed with soap after eating to remove bad smells, chilies, or spices; otherwise, the mothers/other caregivers’ hands would irritate a child’s skin. If a mother cared for her child during her leisure time, she usually did not wash her hands at any of the event identified above, as she perceived her hands as clean. Only two mothers mentioned handwashing as part of a ritual ablution before performing prayers, 5 times each day, customary among many Bangladeshi Muslims.

We observed that washing hands with soap and water was rarely performed [Figs. 2 and 3]. In both sites, handwashing with water alone was most frequently observed before eating, before preparing food for the family, before breastfeeding and after a few events of cleaning the child’s anus and only one event (*n* = 2) after visiting the toilet.

#### Perceived risk of neonatal diseases and perceived advantages and disadvantages of handwashing

In in-depth interviews, few mothers reported perceiving that a child was at higher risk of having illnesses during the first month(s) of life. Their common belief was that the risk is greater during later infancy as the child begins crawling which could increase exposure to illnesses or accidents. As mothers reported that those exposures mainly leading to diarrhea but they did not directly link with other infections. Mothers commonly perceived that neonates are very vulnerable to catching colds (respiratory infection) through ways unrelated to hand hygiene: if the baby is kept in a wet bed soiled with urine or if the mother gets a cold through frequent contact with water or by eating cold food and passing it to her breastfeeding baby through breastmilk. Mothers are also commonly advised to eat warm food and warm water, particularly up to 40 days after delivery to prevent the neonate from getting a cold. Related to these humoral concerns, mothers reported that they avoided frequent contact with water, including decreasing handwashing; mothers avoid handwashing after cleaning a neonate’s anus by wiping with a wet piece of cloth instead of washing the anus by hand. Fathers also reported the similar concern that a mother’s cold can be transmitted to her child. Otherwise, fathers perceived that children get affected by viruses during any seasonal change during the year. However, as we talked, both mothers and fathers did not link the neonate’s ‘cold with ‘respiratory infection’ or other health risks that could lead to neonatal mortality compared to mortality of older infants.

Respondents perceived that cleanliness could protect children from getting ill. Soap was perceived as necessary for cleanliness; it removes visible dirt, germ and bad smells. Mothers reported that they typically wash their hands with or without soap before feeding a child with bare hands, after using the toilet, cleaning a child’s bottom, contact with an older child’s feces, and before preparing food for an infant or family that needs contact with bare hands and is not cooked further (e.g. mashed potato).To mothers, handwashing is important during these events to prevent children or other family members from getting diarrhea from dirty hands. Mothers perceived that, if one held the child with ‘dirty hands’, the child could get diarrhea, skin irritations, cold and fever. Mothers said that ‘visible dirt’ in hands always persuaded them to wash their hands. When hands were ‘visibly dirty’, they reported washing their hands with soap prior to holding a young child to prevent those illnesses. Fathers and others caregivers endorsed the drivers that described above for their or a mother’s handwashing practice. Our observational data also supported this reporting that “visible dirt” (mothers were handling biomass fuel or cooking spices) facilitated required handwashing.

Only one disadvantage of handwashing was cited: one mother in Habigonj stated that if she washes her hands with soap before mashing foods by hand, a common food preparation process in Bangladesh, the mashed item would have a soapy smell.

#### Normative beliefs and subjective norms

We defined subjective norm as the perception that a given behavior is practiced widely in one’s reference network (e.g. neighbors or other mothers of young children). The predominant existing social norms related to childcare contributed to mothers’ normative beliefs. As reflection, mothers performed the expected hygiene behavior for caring her child from the perceived expectation of her close surroundings. According to mothers, feces of an infant who already started eating semi-solid foods including fish and meat, contains germs and smells foul like adult feces; so after defecation or cleaning a child’s anus, they were motivated to wash their hands, mostly with soap, to remove the foul smell from hands and alleviate perceived disgust. In contrast, feces of a young child who breastfeeds, does not contain any germs nor bad smells, and is not ‘disgusting’ to mothers; so handwashing after cleaning the anus of a young breastfeeding child was often perceived as unnecessary.

A normative belief unique to the neonatal period was the possible harmful effect of “bad air” (*alga batash*) or “evil eyes” (evil spirit) during the first 40 to 45 days after birth. All types of respondents typically perceived that an evil spirit could enter the room of an unattended baby and could harm or kill the baby; harm might be reflected in the baby subsequently developing a lack of interest in breastfeeding or having stomach pain. To mitigate this supernatural risk, mothers reported being advised not to go outside, or to leave neonates unattended, especially at mid-day and after dusk. This mobility restriction prevented new mothers’ movement away from the newborn, such that, if the newborn defecated at night, mothers were unable to wash hands because handwashing facilities were typically located outside the sleeping space. This existing normative believe compounded by physical environment that negatively influenced mother’s required handwashing behavior. A mother of a neonate said,

*It is not good to keep the baby unattended in his bed (room) during his first 40 days. Satan may come to the child during this time. It would be better to stay in the post-natal room with the baby....so if required, I wipe up my hands with cloth diaper after cleaning him.*

In Habigonj, we observed two events, when two mothers were about to go outside for handwashing after cleaning a child’s anus but family elders warned mothers about postnatal risk of leaving neonate unattended; as a result, mothers did not go. In both cases, elders were busy with household chores and no one was available to attend the newborn.

However, some social norms also encourage handwashing. Most mothers reported that elder female family members advised and reminded new mothers to wash hands if eating after breastfeeding; otherwise it is believed a mother ingesting her own breastmilk from her hands could cause the death of the child.

Mothers reported that except when elders’ hands were visibly dirty or soiled with dirt, they could not request them to wash hands before holding a child. Mothers generally believed that elders usually do not hold a child with dirty hands.

Many mothers and secondary caregivers perceived that handwashing is not important before holding a child; though it is important before feeding a child. They further explained that a young child is usually wrapped with clothing and does not come into direct contact with one’s hands. Fathers in contrast mentioned that it is important to wash hand first after coming from outside and then hold a young child.

Even though respondents identified the importance of handwashing, they reported not washing hands out of “carelessness” (lack of motivation). One mother of an infant said,

*…from the beginning, Ma advised me to wash hands before breastfeeding…but it (the newborn) breastfeeds frequently… it defecates frequently… (I) wash (only) if hands are visibly dirty, otherwise I do not… I know I should wash hands before every feed…after cleaning (the newborn)…I do not have any problem to wash hand; but I feel idle (lack of motivation/careless) to wash hands every time…I just do not go (for handwash) indolently.*

A number of mothers indicated that, even if they wished to adopt improved handwashing practices during the neonatal period, they would be teased by others. A primiparous mother of neonate said,

*Washing hands before and after every task is not the system (practice) in our place….If I wash hands frequently before or after doing tasks and handling baby, elders (mother in-law/aunts) tease and say, ‘she only has a newborn baby, (as if) we never had’!*

Fathers also added that after coming from outside if anyone wanted to hold a child, it would not possible to ask for a handwash beforehand, otherwise s/he could mind.

While some mothers intended to practice a good handwashing behavior in their daily activities, mothers’ existing habit of handwashing within a certain culture drove them to define when handwashing was required. One multiparous mother of a neonate said,

*I do not wash hands all the time; if my hands are (visibly) dirty, I wash them. If they are less dirty, we wipe them on the door (smiling). We are living in rural area, we cannot do like you people (referring interviewer as urban people); even if you give me so much advice!*

Some mothers, other female caregivers and fathers indicated this habit of infrequent handwashing as a social norm in their rural area, unlike urban culture. One mother of an infant said,

*….(I do not wash hands) because it is not in my habit. If everybody practiced it, I would also follow.*

#### Handwashing as habit

All respondents, regardless of educational and economical status, reported that washing hands with or without soap was habitual only at certain times, such as before and after a meal; they reported developing these habits during early childhood. Despite reported practices, field researchers observed that mothers rarely used soap to wash their hands [Figs. 2 and 3].

#### Nurturing child and maternal intent to improve handwashing behavior

Mothers perceived that a “good mother” always keeps herself and her child clean and soap is a necessary item to maintain cleanliness. One primiparous mother of a newborn who passed sixth grade of schooling said,

*I have to take care of my baby in a good manner... keep him clean (with soap) so that he will get less illness…need to wash him with water after defecation; so he (child) would get less germs. A good mother keeps her child’s clothes, and feeding dishes clean….*

With these characteristics fathers also added that when a child becomes sick, a good mother gives it medicine. Fathers perceived that mother is mainly responsible for nurturing a young child and other family members support her in this process.

Mothers used bar soap (e.g. available brands of antiseptic soap) when bathing or cleaning babies and detergent for washing baby clothes. Mothers underscored that sometimes they might have financial crisis, they always tried to ensure buying soap for a young infant. Only a few mothers said that they could not buy required quantity of soap due to their financial ability. The fathers of young infants and secondary caregivers also mentioned that they know soap is important to keep the baby clean but could not always afford required soaps for their young children due to their financial crisis.

The majority of mothers reported that their handwashing behaviors had increased because of the additional responsibilities of new motherhood, particularly by washing children’s clothes, bathing or sponging the child, washing hands after putting on lotion or an oil massage, after breastfeeding and feeding the infants.

We observed some mothers were simultaneously occupied in multiple chores while caring for their newborns, confirming their reports during interviews that the responsibilities of nurturing a newborn added to regular household chores sometimes prevented mothers from washing their hands. This condition multiplied for mothers who have multiple children. For instance, in the Habigonj site, mothers of multiple children sometimes had to feed the neonate while serving food or cleaning the bottom of an older child after defecation; they often neglected to wash hands after each task. The social construction of woman role being a wife and a mother, often impacted her child nurturing behaviors that restricted her intent to improve handwashing.

#### Maternal self-efficacy and neonatal health protection: Perceived verses actual control over handwashing behavior

Mothers in Matlab reported that they customarily delivered the baby at their parents’ house and that the mother of a newborn was not usually asked to do any household chores when she was at her parents’ house. However, she was expected to perform certain chores at her marital home. During the neonatal period, these mothers often got support from their family for handwashing, e.g. bringing a handwashing station (jug or bucket of water) or soap, or attending to neonates so that the mother could wash hands. Mothers in Habigonj more commonly delivered at their marital home [Table 3]; during neonatal period, if no one was available to help the mother, they brought a female family member to support her in household chores. Conversely, a lack of family support negatively impacted maternal self-efficacy for improving and maintaining good handwashing behavior.

Mothers reported and we also observed in both study sites that when a secondary caregiver was absent, mothers were struggling to carry out varieties household chores and at a time caring a neonate. In such situations, mothers were observed not washing their hands at critical times, for instance after cleaning child’s anus, because the baby became fussy and no one there to attend the baby, mother soothed her child first by breastfeeding. Multiparous mothers in Habigonj who belong to a nuclear family were observed to face this barrier more frequently with the lack of secondary caregivers while they were tending to numerous daily household responsibilities, including caring for neonates and older children.

Many of our mother respondents from both sites reported that if they wished to adopt improved handwashing practices or keep any handwashing materials inside their bedroom during this period for an enabling physical environment, they would be scolded by family elders and neighbors. In this regard, one mother of an infant stated,

*They (in-laws) wouldn’t like it… they wouldn’t allow me to keep it (bucket near bedside)… …They would say ‘they are doing all the tasks and daughter-in-law is not doing anything; she does not even go that little distance (i.e. to the handwashing station)’… she (mother-in-law) will be angry with me.*

Moreover, mothers said that although they often identified items deemed necessary for the child, they didn’t have the power of purchasing materials themselves within existing family roles in social structure. Many of the mothers in Habigonj specifically said that their husbands or sometimes in-laws made the final decisions and purchased related to items needed for childcare. Two mothers of neonates also reported that, when they use too much soap, their husbands scold them as they cannot afford it. Fathers, from the other end confirmed this decision making process and told that they were mainly responsible for outside tasks and also led the treatment related decision (e.g. where to take) when a child got sick.

#### Cues to action: Availability of materials and reminders to wash hands

Mothers in both sites discussed that presence of handwashing materials sometimes facilitated mothers as cues to action for handwashing at times of potential pathogen transmission. When family members supported the mother by bringing required water or soap to her location, she would have been more able to do required handwashing. Sometimes, family members reportedly reminded mothers to wash hands before nurturing. We also observed that when family elders were reminding mothers, mothers washed their hands after household chores and before breastfeeding and holding the child.

Mothers reported and field researchers also observed that, in some cases, mothers avoided some handwashing with their stored water or used only a little water because of the distance to the water source. Some mothers identified lack of soap availability in the places where soap was needed as a hindrance to practicing handwashing. Lack of affordability of soap was also cited as a barrier for some mothers and fathers respondents.

## Discussion

There are sizable opportunities to improve maternal handwashing behavior in the neonatal and infant periods in the Matlab and Habigonj study sites. The lack of handwashing practice during the neonatal period confirms findings from other low-income countries [12, 21]. Consistent with findings from similar formative research in Indonesia, our investigation underscores that the perceived times when caregivers of neonates and infants currently wash their hands are not tightly linked to high risk occasions for pathogen transmission [12]. Existing cultural beliefs related to neonatal health threats, perceptions of disease transmission from caregiver to child, and subjective norms related to handwashing do not align with the biomedical causes, indicating a need to carefully develop behavior change communication strategies as well as designing credible and effective hand hygiene intervention during the neonatal period [22].

A multi-country analysis of drivers of handwashing behavior among mothers of children less than 5 years old has shown that perceptions of risk usually did not motivate maternal handwashing, except in outbreaks such as epidemic cholera [23]. Yet, our study illustrated that mother of a neonate were more concerned and fearful of colds and evil spirits rather than linking to infections during the neonatal stage. Still, mothers in our study reported believing that infants were more vulnerable than neonates, particularly once they began crawling on the ground. This perception related to health threat illustrated that both mothers and fathers were less likely aware about neonatal mortality and its rate compare to infant or childhood mortality. A number of mothers in our study already associated handwashing using soap with diarrhea prevention in reference to infancy and later stages but did not link to prevent transmission of resperitory illnesses. By linking the perceived risks for infants and protective benefit of handwashing in later infancy, awareness could be increased among mothers and other caregivers about the importance of handwashing with soap for reduction of more common neonatal morbidities, such as respiratory or bacterial infections.

Our conceptual model attempted to capture many of the motivators and barriers related to handwashing influenced by physical environment and a number of social dimensions throughout this qualitative research. Some common perceptions in the community, such as elders’ reminders to mothers to wash their hands after breastfeeding to avoid any risk to the child from the mother’s excessive exposure to cold food or cold water [24], are unsound from a biomedical understanding of pathogen transmission. Effective communication within this context requires that we appreciate the prominence of the lay perceptions and identify ways we can connect these perceptions to motivate handwashing behavior in effective and culturally grounded ways that address the emotive drivers likely to benefit the child. Alternative options, such as waterless hand sanitizer, might be considered to minimize the humoral concerns of mother’s excessive exposure to water during the neonate period or during cold winter and to address the challenges of integrating handwashing stations with soap and water in places where mothers are sequestered with newborns. Until the recommended times for handwashing per the biomedical construct align with the caregivers’ understanding in a given cultural context [22], neither their understanding nor their behavior is likely to change.

We found that handwashing appears to be an aspirational behavior rather than a health concern, with some respondents indicating that urban people (connoting a higher level of sophistication) have the habit of washing hands, unlike rural people. The perception of the habit of handwashing is critical to behavior change in other settings and can be deeply embedded in culture [23]. We found through observation and self-reporting that having a handwashing habit is not the norm in these communities. Mothers in our study connected a number of existing beliefs and social norms as drivers of handwashing behavior but rarely for health concerns for the child until hands were visibly dirty [23, 25]. Even if mothers intended to improve handwashing behavior during the neonatal period, physical environment or constructed attitude to handwashing and existing dimensions in social relations hindered their practice. Their intent to wash hands was sometimes counterbalanced by the lack of support from family members for changing the social norm, the substantial increase in workload faced by mothers of newborns, and the lack of agency for some to make newborn-related household purchasing decisions. Furthermore, fathers’ perceived notion to mother, as prime nurturer of child might not incite them to guide mothers for a better hand hygiene. With this existing construction of woman’s role, the pattern of delivery in marital home and conservative social structure likely to put mother at Habigonj during neonatal period in trickier situation to execute required hand hygiene.

These factors suggest that interventions that promote handwashing to mothers of neonates or young infants need to address the broader context by engaging her family and community to make her physical and social environment conducive to handwashing. Focused handwashing instructions on a few specific key times that represent the greatest risk of pathogen transmission may be more practical for mothers to follow in their busy daily schedule. The focused handwashing instructions also need to be streamlined with caregivers’ cultural context and biomedical context with credible interventions, such as waterless hand sanitizer option to minimize time constrain to mothers of neonates.

We observed that the availability of handwashing materials where the mother-baby dyad are located (particularly during mothers’ restricted mobility in first 40 days of baby’s life) and reminders from elders to wash hands before nurturing a baby facilitated mothers’ handwashing. However, mothers noted that a simplistic approach of just providing a handwashing station will not overcome all barriers. There must be buy-in from elders who otherwise may scoff at the notion of handwashing before holding the baby, or who may ridicule the mother for prioritizing materials to facilitate her child-rearing. Although this model does not directly mention poverty or lack of agency of mothers to secure soap and a handwashing station, affordability and supply of materials were cited as important barriers in many households of similar settings [4]. Therefore, a combined approach included both the existing social norms and a dedicated handwashing device with storage of water and soap during certain stages of a baby’s life may create an enabling environment for mothers to perform suggested handwashing behaviors. Considering the social limits of the mother’s role, major decision makers or influential members within the family (e.g. husband, mother in-law) need to be engaged in hand hygiene interventions, to follow the suggested hygiene practice, or to establish a new, temporary and convenient handwashing station. For this initiative, health workers’ involvement could be included for the communication of information related to neonatal health threats and clarifying the prevention measures to mothers and family members during an antenatal visit in the local healthcare facilities. Local birth attendants might also be considered to convey the information to mothers and female elders who receive homecare.

One of the limitations of our study is the possibility of courtesy bias during observation. While direct observation is commonly used to measure hand hygiene, the presence of an observer has been shown to increase handwashing behavior in another study in Bangladesh [26]. Participants may have reacted to the presence of the observers by increasing handwashing [27]. Despite any possible reactivity, there was very little handwashing with soap, suggesting that the observed lack of habitual handwashing with soap is accurate or even worse than observed [26, 28]. Another possible limitation is conducting the observations in both sites during the winter season, when mothers may wash hands less frequently than during other seasons due to humoral concerns related to water contact and the risk of respiratory infections. However, similar handwashing rates were reported in other available studies conducted in other seasons [10].

## Conclusions

This qualitative study identified a number of important barriers and facilitators to handwashing behavior among mothers and other caregivers of neonates and young infants in the complex social fabric of Matlab and Habigonj, Bangladesh. Designing context-specific interventions based on the dominant social norms and contextual barriers and facilitators identified in our study, rather than blanket approaches based on health messaging that principally targets mothers to the exclusion of other family actors, may yield interventions that are more effective at motivating handwashing behavior change.

## Additional file

Additional file 1: Guideline for semi-structured observation, The semi-structured observation data collection guideline has been included to better understand how the data was collected and therefore results were presented in Figs. 2 and 3. (DOCX 22 kb)

## Abbreviations

BDHS: Bangladesh demographic and health survey
HDSS: Health and demographic surveillance system
icddr,b: International centre for diarrhoeal diseases research, Bangladesh
MaMoni: Integrated safe motherhood, newborn care, and family planning
MCH: Maternal and child health
USAID: United States agency for international development
