# Supplementary material for: Barriers to and motivators of handwashing behavior among mothers of neonates in rural Bangladesh
Source: BMC Public Health. 2018 Apr 11;18:483. doi: 10.1186/s12889-018-5365-1 (PMC5896121; doi:10.1186/s12889-018-5365-1)
Supplement: Supplementary file 1 — Guideline for semi-structured observation, The semi-structured observation data collection guideline has been included to better understand how the data was collected and therefore results were presented in Figs. 2 and 3. (DOCX 22 kb) [file 12889_2018_5365_MOESM1_ESM.docx]

**Additional file 1**

**Guideline for semi-structured observation**

Title of the manuscript: Barriers to and motivators of handwashing behavior among mothers of neonates in rural Bangladesh

Participant ID#:

Name of the Participant _______________________

Date of Observation: / /

Start time (24 hr, HH:MM): End Time (24 hr, HH:MM):

Objective

To gather a full description of the environmental experience of a new mother, in order to more fully understand the challenges and opportunities for introduction of a handwashing intervention in these women

**Background information:**

- Family member, Family type:

- Description of the household:

- Economical background of household:

- Description of the mother:

- Description of the child:

**Instructions:**

Please describe- the circumstances and environment the mother experiences before, during, and after critical times for handwashing. We are interested in structures, reminders, concurrent events, or conditions that make it easier or harder for the mother to wash her hands. Record as much information as possible even if you do not think it is important, as well as overall impressions of the event and subsequent handwashing action or inaction. Additionally, include overall observations of the general household structure and environment which may facilitate or prevent handwashing behavior. The time may either be a notation of the time of the critical event, or the time you observe a facilitating or prohibitive behavior or structure. The observation period is 5 hours. If it is necessary to step away from the observation, record departure and return times on the observation record.

**Suggestions of information to include with environment assessment (checklist):**

- How is the mother’s day structured?
- In the time around critical events for handwashing, what was mother experiencing? Was she washed her hands? What allowed her to do this? Was there water nearby; was she already near a handwashing area? If she was unable to wash her hands, what do you see that may have prevented her from doing this? Was she holding the baby, was she far away from soap and/or water at the time?
- Was the mother attentive to the baby? What nurturing behavior did she display and when?
- Who else was present in the home? Are they assisting with care of the baby? Who appeared to have control over how the baby is cared for?
- Was water available? How often did the mother have to get water and where did she get it from? Where was the water stored?
- Critical handwashing opportunities to assess.
- After cleaning the child’s anus
- After defecation (self)
- Other fecal contact (other children, animal, etc)]
- After contact with respiratory secretions of the child (cough or sneeze, wiping nose) or self
- Before preparing food
- Before feeding the infant
- Before eating
- Serving food to the family members

##### OBSERVATION RECORDING FORM

Name of the Observer: Time observation begins (24 hr, HH:MM):

Name of Mother: Time observation ends (24 hr, HH:MM): Date:

Name of Child:

Age of Child:

| Time | Critical times/events | Does she wash hands? With water/soap/others? | Is there any motivator? | Is there any barrier? | Anyone assist for caring baby/any helper |
| --- | --- | --- | --- | --- | --- |
|  |  |  |  |  |  |
|  |  |  |  |  |  |
|  |  |  |  |  |  |
|  |  |  |  |  |  |
|  |  |  |  |  |  |
|  |  |  |  |  |  |
